# P-Element Homing Is Facilitated by *engrailed* Polycomb-Group Response Elements in *Drosophila melanogaster*

**DOI:** 10.1371/journal.pone.0030437

**Published:** 2012-01-19

**Authors:** Yuzhong Cheng, Deborah Y. Kwon, Allison L. Arai, Diane Mucci, Judith A. Kassis

**Affiliations:** 1 Program in Genomics of Differentiation, Eunice Kennedy Shriver National Institute of Child Health and Human Development, National Institutes of Health, Bethesda, Maryland, United States of America; 2 Division of Cellular and Gene Therapies, Center for Biologics Evaluation and Research, Food and Drug Administration, Bethesda, Maryland, United States of America; The University of Chicago, United States of America

## Abstract

P-element vectors are commonly used to make transgenic Drosophila and generally insert in the genome in a nonselective manner. However, when specific fragments of regulatory DNA from a few Drosophila genes are incorporated into P-transposons, they cause the vectors to be inserted near the gene from which the DNA fragment was derived. This is called P-element homing. We mapped the minimal DNA fragment that could mediate homing to the *engrailed/invected* region of the genome. A 1.6 kb fragment of *engrailed* regulatory DNA that contains two Polycomb-group response elements (PREs) was sufficient for homing. We made flies that contain a 1.5kb deletion of *engrailed* DNA (*en^Δ1.5^*) *in situ*, including the PREs and the majority of the fragment that mediates homing. Remarkably, homing still occurs onto the *en^Δ1. 5^* chromosome. In addition to homing to *en*, *P[en]* inserts near Polycomb group target genes at an increased frequency compared to *P[EPgy2]*, a vector used to generate 18,214 insertions for the Drosophila gene disruption project. We suggest that homing is mediated by interactions between multiple proteins bound to the homing fragment and proteins bound to multiple areas of the *engrailed/invected* chromatin domain. Chromatin structure may also play a role in homing.

## Introduction

P-element mediated transformation has been used to generate transgenic Drosophila for about 30 years [1]. In large-scale screens, P-element based vectors have been used for mutagenesis and for enhancer-detection [2]–[6]. In these cases, P-element vectors were found to insert in the genome in a relatively non-selective manner, with the exception of some hotspots for insertion. However, in a few specific cases, when particular fragments of regulatory DNA are included in a P-element vector, they dramatically alter the insertional specificity of that vector. The first case of this was described by Hama, Ali, and Kornberg in 1990 [7]; regulatory DNA from the *engrailed* (*en*) gene, when included in a P-element vector (*P[en]*), caused *P[en]* to insert in the vicinity of the *en* gene at a high frequency. They named this phenomenon P-element homing. Since that time, regulatory DNA fragments from the *linotte* gene, *bithorax* complex, and the *even-skipped* (*eve*) gene have been found to mediate homing [8]–[10]. For homing mediated by *en*, *bithorax*, and *eve* DNA, insertions are not site-specific but are regional; that is, they occur in the vicinity of the endogenous gene but can be distributed over a large genomic region. For homing by *P[en]*, insertions occur over about a 300 kb region, including *en*, *inv* and flanking genes [7], [11], [12].

The segmentation gene *en* exists in a gene complex with the related gene *invected* (*inv*). *en* and *inv* encode homeodomain-containing proteins, are co-expressed, and share regulatory DNA [13]. *en* and *inv* are regulated by the Polycomb group genes (PcG) [14]. In tissue culture cells and embryos, H3K27me3, the distinctive PcG chromatin modification, covers a 100kb region that includes *en* and *inv*, but not flanking genes [15]. In chromatin immunoprecipitation experiments in tissue culture cells and adults, there are three regions of *en/inv* that associate with PcG proteins, one just upstream on the *en* promoter, one coincident with an *inv* promoter, and one 6 kb upstream of *inv* [15], [16]. These fragments of DNA act as Polycomb group response elements (PREs) in transgenic Drosophila [17]–[19]. The PRE located upstream of the *en* gene is a complex element, including 2 minimal PREs [18], [20].

We have been using P-element homing by *en* DNA as a tool for our experiments for about 20 years. For example, we used a homing P-element to insert a mosquito *en* cDNA onto an *en* mutant chromosome to show it can substitute for the Drosophila *en* protein coding region [11]. More recently, we used *P[en]* homing to generate a large number of *P[en]* insertions near *en* and study the long-range action and promoter specificity of *en* enhancers [12]. Here we took two experimental approaches in order to further understand the mechanism of *P[en]* homing: 1) we dissected the *en* homing fragment and 2) we deleted the homing fragment from the genome, and asked whether homing could still occur. Our data show that the minimal fragment of DNA that can mediate homing is 1.6kb. Since we could not get a smaller fragment of DNA to mediate homing, we suggest that homing is not caused by a single protein or protein complex. Second, we found that homing still occurs, albeit at a reduced frequency, into genomic DNA that lacks the majority of the homing fragment. Therefore, homing is not solely dependent on interactions between the genomic and *P[en]* homing fragments. Our data suggest that homing is mediated by multiple protein-protein interactions between proteins bound to the homing fragment in *P[en]* and proteins bound to multiple regions of the *en/inv* genes. Since homing occurs in the germ line, we suggest that the *en/inv* genomic region is bound by many regulatory proteins and has a specific chromatin structure in germ cells.

## Results

### Identifying a minimal homing fragment

**Figure 1:**
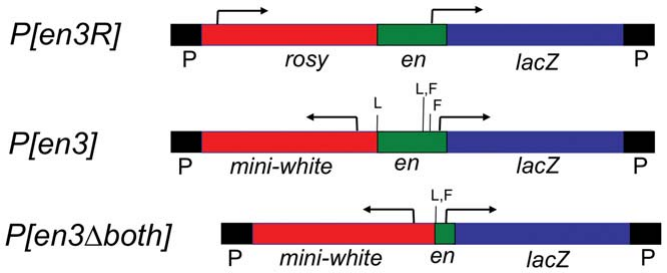
P-constructs used in homing experiments. Arrow shows the direction and start of transcription. L, loxP site. F, FRT site. *P[en3R]* is from [21] (called *P[en1]* in that study). *P[en3]* and *P[en3Δboth]* are from [18].

**Figure 2:**
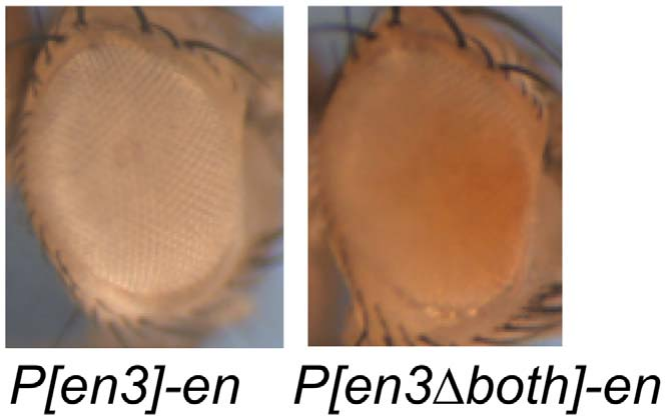
PREs silence mini-*white* expression in *P[en3]*-*en* eyes. Eyes of 5-day old male flies are shown. *w; P[en3]-en/+* (left) and *w; P[en3Δboth]-en/+* (right).

In previous experiments, we found that a 2.6 kb DNA fragment, extending from −2.4 kb to +188 bp was sufficient to mediate homing to the vicinity of the *en/inv* region of the genome at a frequency of 5% (7 out of 131 insertions; [21]) (Construct *P[en3R],* Fig. 1, called *P[en1]* in [21]). Since that time, whenever this *en* fragment was included in a P-element vector we localized the P-insertions in the genome. In 2002, we studied the pairing-sensitive silencing properties of the 2.6kb fragment, and generated 43 lines, using mini-*white* as the reporter gene to recognize transgenic flies [17]. Insertions into the vicinity of *en/inv* region occurred at a frequency of 4.6% (2/43; Table 1). In another study, we generated 21 lines using *P[en3]* (Fig. 1), and obtained two insertions in the vicinity of *en/inv* (a frequency of 10%; [18]). In *P[en3]*, the reporter gene used to recognize transgenic flies is mini-*white*. Notably, flies that contained *P[en3]* inserted 6kb upstream of *en* (*P[en3]-en*) had white eyes (Fig. 2). This line was recovered fortuitously; when using inverse PCR to localize the site of a *P[en3]* insertion, we identified two insertions in one transgenic fly, one on chromosome 3R, and the other at *en* (*P[en3]-en*). The white eye color of *P[en3]-en* is due to the repression of mini-*white* by the PREs in the 2.6kb fragment, since deletion of both PREs by Cre recombinase and Flp recombinase to generate *P[en3Δboth]* gives flies with colored eyes (Fig. 2; no eye color was seen when either one of the PREs was present). In contrast, flies with *P[en3]* inserted into *tou*, the gene just upstream of *en,* have light orange eyes (not shown). Using constructs similar to *P[en3]*, but with different reporter genes (an *en-lacZ* fusion protein, construct F in [22]; the mosquito *en* cDNA [11]) we know that insertions into the *inv* intron and just downstream of the *en* transcription unit give flies with colored eyes. Nevertheless, because mini-*white* silencing by the PREs would lead us to miss insertions in the vicinity of the *en* promoter, the homing frequency obtained in experiments using *P[en3]-*mini-*white* vectors is probably an underestimation of the true homing frequency.

**Table 1:** Insertions near *en/inv* on wild-type chromosomes.

Construct	Line^a^	Position^b^	Gene^c^	Location^d^	Reference
*2.6lacZ*	2A	−751 bp	*en*	7416139	17
*2.6up*	4A	−87 kb	*tou*	7492660	17
*P[walter]*	48A	−67 kb	*tou*	7482784	26
*P[walter]*	4A	−86 kb	*tou*	7501690	This study
*P[en3]*	*en*	−6 kb	*en*	7421628	18
*P[en3R]*	1A	−472 bp	*en*	7415760	This study
*P[en3R]*	11A	−72 kb	*tou*	7487611	This study
*P[en3R]*	+52kb	+52 kb	*inv*	7363188	12
*P[en3R]*	−87kb	−87 kb	*tou*	7502613	12

a Line names

b The number of kilobases or base pairs upstream (negative numbers) or downstream (positive numbers) of the major *en* transcription start site (at 7415388) [35].

c Closest gene.

d Nucleotide insertion site (genome version R5.1, FlyBase).

**Figure 3:**
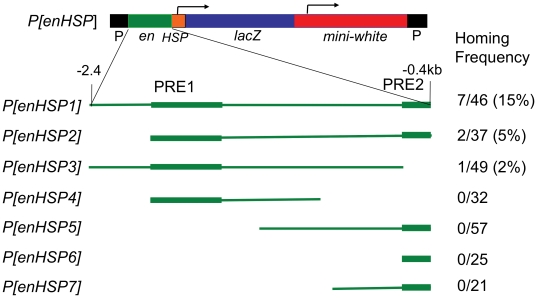
*P[enHSP]* constructs used to dissect the homing fragment. The *P[enHSP]* construct is shown on top with the extent of the *en* DNA present shown below by the green line. The PREs are shown as thicker areas of the green line. The homing frequency is shown on the left; the number of insertions in the *en/inv* region/the total number of transgenic lines recovered is shown on the right side. *P[enHSP1]*, *P[enHSP2]*, and *P[enHSP3]* are from [12].

Experiments designed to test PRE activity lead us to use the vector *pUZ* [23]. In this vector, the mini-*white* promoter is separated from the PRE by the *lacZ* transcription unit (Fig. 3). We made a *pUZ* construct containing a 2 kb *en* fragment, extending from −2.4 to −0.4 kb upstream of the *en* transcription unit (*P[enHSP1]* [12]; Fig. 3). Notably, when *P[enHSP1]* was inserted in the genome just upstream of *en*, the flies had colored eyes (not shown). The homing frequency of *P[enHSP1]* was 15% (7/46 lines; one line had two insertions, one in *en* and one in *inv*, this line was only counted once). Similar to the previous experiments, *P[enHSP1]* insertions were not site specific but occurred over a 149kb region with two insertions in *en*, two in *inv*, three in *tou*, and one in *E(Pc)* [12].

We used the *pUZ* vector to narrow down the DNA fragment required for homing. Deletion of the 181bp *en* PRE decreased the homing ability of this fragment (*P[enHSP3]*) when compared with *P[enHSP1]* (2% versus 15%, P = 0.0274 by a Fisher's exact test). Therefore we conclude that the 181bp fragment contributes to homing efficiency. Can the 181bp-*en* PRE alone mediate homing? Over the years we have generated 166 lines with the 181-bp fragment cloned upstream or downstream of mini-*white* in various constructs ([17], [20] and *P[enHSP6]* in Fig. 3) and mapped their insertions sites using either recombination mapping, hybridization to polytene chromosomes, or inverse PCR, and never recovered an insertion near the *en* gene. Also, in this study, *P[enHSP5]* and *P[enHSP7]* contain the 181-bp fragment and we did not obtain any inserts of these two constructs into the vicinity of *en/inv*. Thus we conclude that the 181-bp fragment is not sufficient for homing to *en*.

Data from a 1994 study [20] led us to test the ability of the fragments in *P[enHSP4]* and *P[enHSP5]* to home. In that study, one P-insertion from a *pCaSpeR* construct with sequences extending from −1184 to −944 bp was localized to polytene band 48A on salivary chromosomes, out of 4 insertion events (its exact location was never determined and the line was lost many years ago). Second, we obtained an insertion of a *pCaSpeR* construct containing sequence −1944 to −1166 bp into *E(Pc)*, out of a total of 13 insertion events. Both *P[enHSP4]* and *P[enHSP5]* contain the fragment extending from −1184 to −944 bp. Out of 89 lines obtained, none was inserted in the vicinity of *en*. *P[enHSP4]* contains the sequences −1944 to −1166 bp. We did not obtain any insertions into *en* in the 32 lines recovered. Thus, our results suggest that if these fragments cause homing, it is at a very low frequency.

In this study we did find one sub-fragment that mediates homing. *P[enHSP2]* contains a 1.6kb fragment that includes the two PREs. Two out of 37 insertions of this construct were at *en* (5%). The homing frequency of *P[enHSP2]* is not significantly different from that obtained with *P[enHSP1]* (P = 0.2867 by a Fisher's exact test). Therefore, we consider the 1.6kb fragment to be the minimal homing fragment.

### Homing occurs in flies that lack the genomic homing fragment

**Figure 4:**
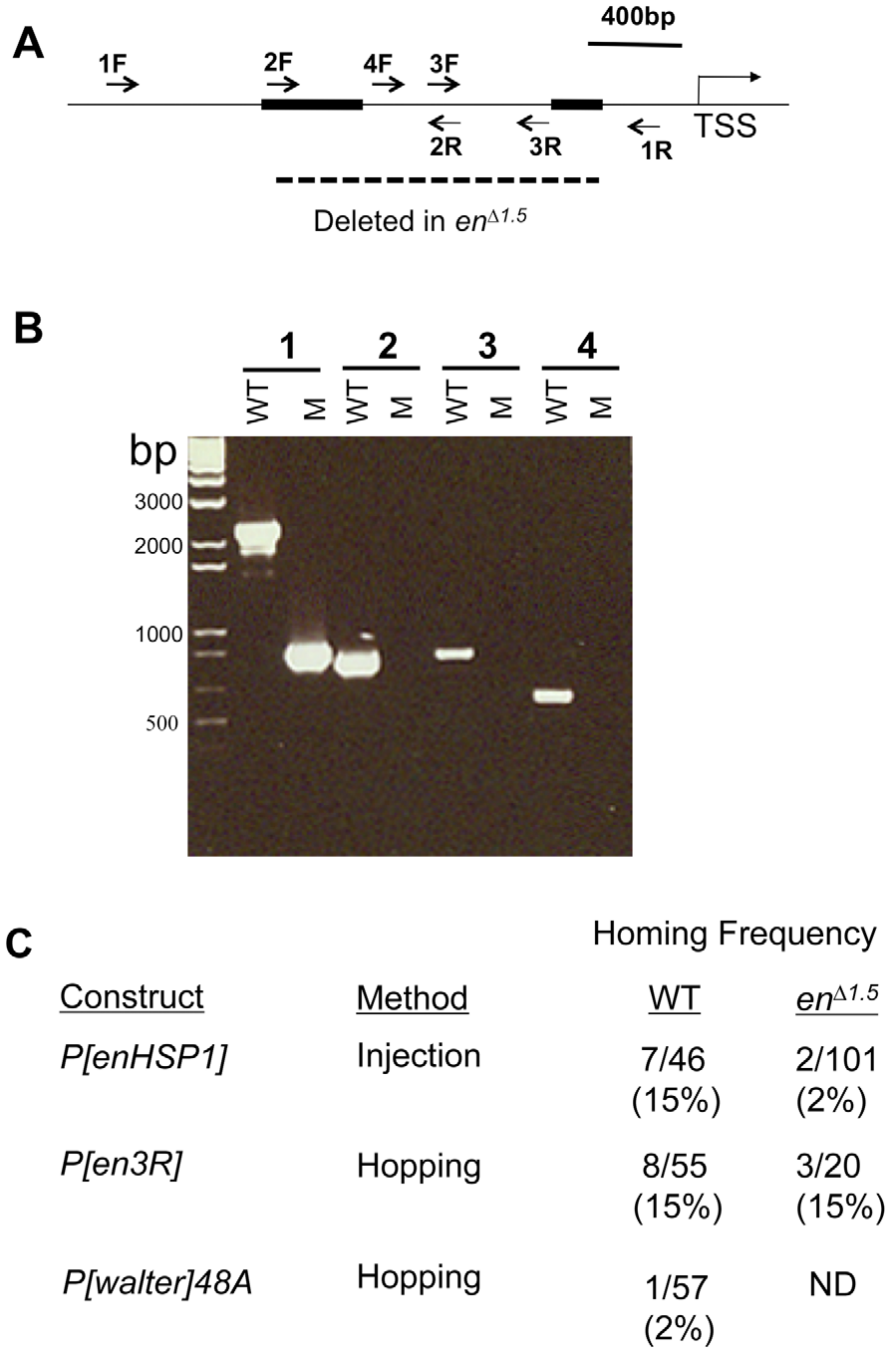
Characterization of and homing to the *en^Δ1.5^* chromosome. (**A**) Thin line represents DNA upstream and including the transcription start site (TSS) of *en*. PRE1 and PRE2 are depicted by rectangular boxes. The primers used for PCR reactions are indicated. The extent of the *en^Δ1.5^* deletion is indicated by the dashed line. (B) PCR reactions using wild type (WT) or *en^Δ1.5^* DNA (M) and the following primer sets (1) 1F and 1R (2) 2F and 2R (3) 3F and 1R (4) 4F and 3R. The M PCR product from primer set 1 was sequenced and shows that 1462 bases are deleted in *en^Δ1.5^*. (C) Homing frequency onto wild type and *en^Δ1.5^* chromosomes. Homing frequency is the number of insertions near the *en/inv* region/the total number of transgenic lines. ND, not done.

We generated a 1.5kb genomic deletion that removes both PREs upstream of the *en* transcription start site (Fig. 4A, B), *en^Δ1.5kb^*. These flies are both viable and fertile, and have a loss-of-function wing phenotype similar to that of *en^Δ530^*, which contains a smaller deletion in the same region [18]. *P[enHSP1]* was injected into *en^Δ1.5kb^* homozygous embryos; two insertions (out of 102) were in the *en/inv* chromosomal region; one insertion was into the *en* promoter, and one was in the *inv* promoter (Table 2, Fig. 4C). The frequency of homing into *en^Δ1.5^* flies was reduced as compared to wild-type (2% vs. 15%; P = 0.0041 by a Fisher's exact test), however, it is notable that it occurred at all, given that majority of the fragment required for P-element homing was deleted from the endogenous *en* gene in these flies.

**Table 2:** Insertions near *en/inv* on chromosomes carrying *en^Δ1.5^*.

Construct	Line^a^	Position^b^	Location^c^
*P[en3R]*	*inv*	+52 kb	7363188
*P[en3R]*	*en*	−216 bp	7415604
*P[en3R]*	*CG9005*	−105 kb	7520040
*P[enHSP1]*	*inv*	+52 kb	7363272
*P[enHSP1]*	*en*	−26 bp	7415414

a Lines are the named by the closest gene. All lines were generated for this study.

b The number of kilobases or base pairs upstream (negative numbers) or downstream (positive numbers) of the major *en* transcription start site (at 7415388) [35].

c Nucleotide insertion site (genome version R5.1, FlyBase).

As an additional test as to whether homing could occur onto *en^Δ1.5^*, we mobilized *P[en3R]-87kb*, an insertion in the *tou* gene on a *CyO* chromosome with *P(Δ2,3)* transposase [24] and screened for transposition off the *CyO* chromosome onto the *en^Δ1.5^* chromosome. Of 16 transposition events, two occurred into the vicinity of *en/inv*; one 1.2kb downstream of the *inv* promoter, and another insertion 20kb upstream of *tou* (a homing frequency of 12.5%) (Table 2). Mobilization of *P[en3R]+52kb*, inserted in *inv* on a *CyO* chromosome also generated an insertion on *en^Δ1.5^* near *en/inv*, upstream of *CG9005* (out of 4 transposition events, Table 2, Fig. 4C). As a control we mobilized *P[en3R]-87kb* with *P(Δ2,3*) transposase in a wild-type 2^nd^ chromosome background and obtained one insertion into the *tou* gene and another insertion 55 bases upstream of the *en* transcription start site (2/21 transposition events, a frequency of 9.5%, Table 1; Fig. 4C). In another experiment we mobilized a different *P[en3R]* insertion in *tou* to generate 34 transposition events in a wild-type 2^nd^ chromosome background and 6 were in the vicinity of *en/inv* (17%). Thus, by P-element mobilization, the frequency of homing is similar onto a wild-type and *en^Δ1.5^* chromosome.

P-elements are known to transpose locally, and, in one study, it was reported that there was also preferential transposition of P-elements to a corresponding region of the homologous chromosome [25]. Therefore we wondered whether the high frequency of transposition events obtained from *P[en3R]* inserted into *tou* and *inv* were the result of local transposition or representative of homing to the *en/inv* region. Of note, *CyO* does not disrupt homolog pairing in the vicinity of *en/inv*, thus, local transposition was a distinct possibility. We tested whether there is preferential transposition to a corresponding region of the homologous chromosome in the vicinity of *en/inv* using a *P[walter]* element inserted in *tou* (Table 1). *P[walter]-48A* contains a modified mini-*white* gene used to detect gene conversion events [26] and does not contain a PRE. Thus, by analogy with *P[en3]Δboth*, mini-*white* would not be expected to be silenced if *P[walter]* is inserted in the vicinity of *en/inv*. We looked for transposition of *P[walter]-48A* off a wild-type chromosome onto either a *CyO* or a chromosome carrying the marker *Sp*, but wild type for the *en/inv* region. 52 *P[walter]* transposition events were found, and only one was near *en*, inserted in *tou*, a frequency of 1.9% (1/52). This is much lower than rates obtained by transposition of *P[en3R]* onto either a wild-type (8/55) or *en^Δ1.5^* chromosomes (3/20) (P = 0.0126 by a Fisher's exact test compared to wild-type; P = 0.0516 compared to *en^Δ1.5^*). We suggest that the high rate of insertion into the *en/inv* region via transposition from *P[en3R]* inserted in *tou* or *inv* is largely the result of homing and not local transposition.

### Are there other preferred targets for *P[en]* constructs?

To address the question of whether constructs containing the homing fragment have additional preferred targets, we analyzed the insertion sites of all 40 *P[enHSP1]* insertions generated by injection into *en^Δ1.5^* embryos that mapped to chromosome 2 (Table 3). We reasoned that the *en^Δ1.5^* chromosome should not alter the chromatin structure of areas outside the *en/inv* region of the chromosome. In this experiment, 40 of 102 *P[enHSP1]* insertions were on chromosome 2, including one at the *en* and one at the *inv* promoter (Table 3). We recovered four insertions into *lola*, a hotspot for P-element insertions, two into *bun*, another P-element hotspot, showing that *P[enHSP1]* behaves like other P-elements with respect to P-element hotspots [3]. In addition, we obtained two insertions into the gene *Vha16-1* (Table 3). *Vha16-1* is not a reported hotspot for P-element insertions although there are 29 P-element inserts in this vicinity recorded in FlyBase. From these data we conclude that *P[enHSP1]*, aside from it's homing to *en/inv* region, behaves like most other P-elements in its target site selection.

**Table 3:** *P[enHSP1]* insertions on chromosomes carrying *en^Δ1.5^*.

Line^a^	Location^b^	Gene^c^
5A	2L:109235	*Sam-S*
f-14-3	2L:11057544	*Samuel*
f-11	2L:11173008	*Vm32E*
16A	2L:12011113	*Rha5, CG6734*
m-55-L	2L:12539996	*bun* *
m-58	2L:12540005	*bun* *
4E	2L:14234118	*smi35A*
f-29V	2L:14490554	*noc*
m-55-S	2L:14997589	*mol*
**f-89**	**2L:16485712**	***dac***
f-14-1	2L:16685386	*grp* *
f-28-1	2L:20382385	*CG16798*
10A	2L:20972144	*CG42238*
2B	2L:21618794	*CG2201*
**4D**	**2L:21828581**	***tsh***
**2A**	**2L:22019207**	***CR33987***
6A	2L:5108441	*Msp-300*
30f1	2L:5981758	*eIF-4A*
14C	2L:8416598	*Akap200*
m-98	2R:12468461	*Cdk4*
m-69	2R:12744739	*Dek*
**2C**	**2R:1632293**	***ap***
f-23	2R:18100371	*ari-2*
m-3-1	2R:19757865	*ken*
m-53-L	2R:20289791	*slik*
12A	2R:20857342	*CG3776*
f-28-2	2R:221423	*Gprk1*
m-47	2R:2518935	*Vha16-1*
m-45	2R:2519424	*Vha16-1*
m-74	2R:2839867	*mim*
f-58	2R:2993199	*esn*
14E	2R:4061646	*CG14757*
15C	2R:6421820	*lola* *
14B	2R:6422854	*lola* *
3A	2R:6429207	*lola* *
15A	2R:6429232	*lola* *
***inv***	**2R:7363272**	***inv***
***en***	**2R:7415414**	***en***
6B	2R:7779622	*E1alpha48D*
f-12	2R:8481109	*Amph*

a Name of line.

b Nucleotide insertion site (genome version R5.1).

c Nearest gene(s).

*Hotspot for P-element insertions [3]. PcG targets are shown in bold.

It has been suggested that P-elements that contain PREs insert in the genome near PcG-regulated genes (reviewed in [27]). Therefore, we examined if any of the *P[enHSP1]* insertion sites were in PcG targets. For PcG targets we used the class I high confidence target genes from Schwartz et al., 2010 [28]. Insertions of *P[enHSP1]* were found in 6 PcG target genes: *CR33987*, *apterous*, *teashirt*, *dachshund*, *en*, and *inv*. Notably, these genes are not hotspots for P-element insertions.

It was recently reported that Polycomb-target genes are cold spots for P-elements and other transposons [4]. In that study, the sites of 18,214 unselected *P{EPgy2}* insertions were determined [4]. Of these 18,214 insertions, only 156 were into class I high-confidence PcG targets, including 62 in *apt*, and 20 in *esg*, both P-element hotspots. There are no *P{EYgy2}* insertions into *en*, *inv*, or *dachshund*. The number of *P[enHSP1]* insertions in PcG targets (6/102) is significantly different than the number of *P{EYgy2}* (156/18,214) (P<0.0001, chi-square test). Thus, our data is consistent with a model that P-elements that contain PREs have an increased frequency of insertion into PcG-regulated genes.

## Discussion

### A 1.6kb fragment of *en* DNA can mediate homing

Our previous results indicated that a 2.6 kb fragment of *en* DNA, extending from −2.4 kb upstream through +188bp of the *en* transcription unit could mediate P-element homing to the *en/inv* domain [21]. Here we show that a 1.6kb fragment that extends from −2.0 kb through −0.4 kb is sufficient for homing. We had hoped to identify a small fragment of *en* DNA that could mediate homing, but this was not the case. Thus we suggest that homing is mediated by a complex array of proteins and/or chromatin structure.

PcG proteins are thought to mediate long-range chromatin interactions at the Bithorax complex [29], between the Bithorax and Antennapedia complexes [30], and also between PcG targets on the same chromosome arm [31]. We note that one study suggests that the interactions at the Bithorax complex are not mediated by PREs, but by closely associated insulator elements [32]. Biochemical studies show that PcG protein complexes can interact in vitro [33]. Our results suggest that PREs play a role in *P[en]* homing: 1) deletion of the 181-bp PRE in the transgene decreases the homing frequency and 2) *P[enHSP1]* insertions occur in PcG-regulated genes at a higher frequency than *P{EYgy2}* insertions. We note that both the *eve* and Bithorax homing fragments are thought to be insulator elements [9], [10]. The *en* homing fragment is located just upstream of the *en* promoter and we consider it unlikely that it is an insulator. However, the insulator proteins GAGA Factor, CTCF, and Mdg4 are associated with this DNA in embryos [15]. Therefore, it is possible that the homing fragment has some of the same properties as insulators.

In our previous study [21], we found that embryonic *lacZ* expression from *P[en3R]* (called *P[en1]* in that study) occurred in stripes at a much higher frequency than with the enhancer trap *P[lacW]*. Our hypothesis was that *P[en3R]* caused selective insertion of *P[en3R]*, not just to *en/inv*, but also to many genes expressed in stripes. We know now that both the *en* promoter and *en* PREs (or sequences closely associated with them) mediate interactions with distant enhancers [12], [18]. Thus, one reason for the enriched number of *lacZ* stripe patterns with *P[en3R]* could be its ability to work with distant enhancers. In support of this, when *P[en3R]* is inserted up to 140 kb and 5 transcription units away from the nearest *en* stripe enhancer (either upstream or downstream), *P[en3R]*-encoded *lacZ* is still expressed in *en*-like stripes [12]. In contrast, when *P[lacW]* is inserted about 45kb upstream of the nearest *en* stripe enhancer, into *tou*, the gene adjacent to *en*, *P[lacW]*-encoded *lacZ* is not expressed in stripes [12]. In fact, the PREs in *P[en3]* facilitate long-distance interactions with enhancers in many different regions of the genome [18]. We suggest that the high percentage of striped *lacZ* expression from *P[en3R]* insertions is due both to the ability of the *en* promoter and PREs to act with distant enhancers and also to increased insertion into PcG-regulated genes, many of which are developmental regulators and expressed in stripes.

### Homing in *en^Δ1.5^*

We generated a 1.5 kb deletion of *en* DNA *in situ*, including the two PREs. Surprisingly, *en^Δ1.5^* flies are homozygous viable and fertile. En expression appears normal in these flies (data not shown). We suggest that these PREs are redundant with *inv* PREs and that the *en/inv* H3K27me3 domain is not disrupted in *en^Δ1.5^* flies. Interestingly, *P[en]* homing still occurs in *en^Δ1.5^* flies. These data suggest that homing is not mediated solely by self-self interactions between the homing fragment in *P[en]* and the genomic homing fragment.

### A model for *P[en]* homing

**Figure 5:**
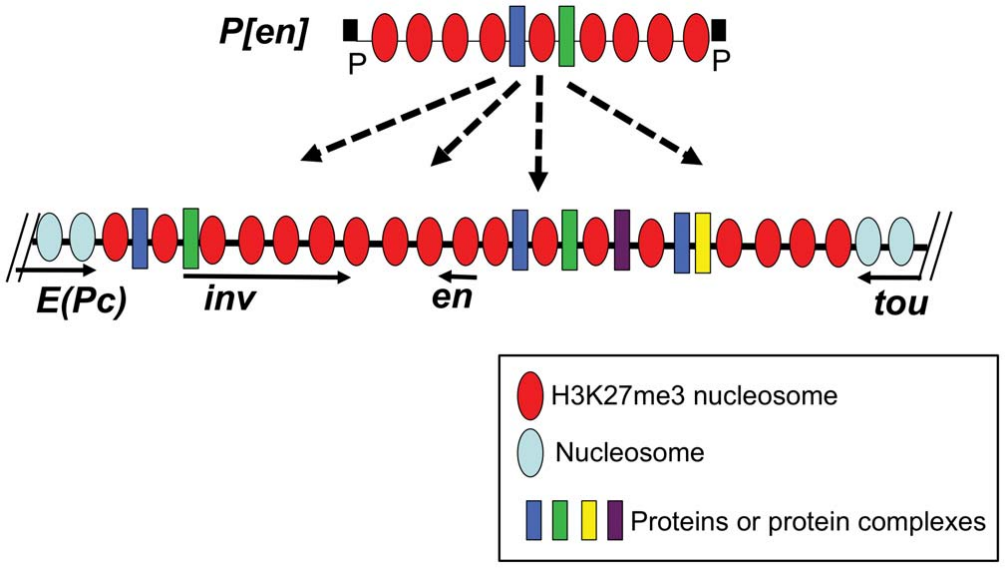
A model for *P[en]* homing. *P[en]* and the *en/inv* DNA are packaged in H3K27me3 nucleosomes (red ovals) [15]. Nucleosomes associated with *tou* and *E(Pc)* do not have the H3K27me3 mark (blue ovals) [15]. Specific proteins bound to both *P[en]* and *en/inv* DNA are represented by different colored rectangular boxes. We suggest that the *en* DNA in *P[en]* adopts the same chromatin structure and has the same proteins bound to it as in the genomic context. (*P[en]* is not drawn to scale). Since the *inv* promoter is co-regulated with *en*, and also has a PRE associated with it, we draw its structure as identical to the *en* promoter. We suggest that P-element homing occurs through specific interactions between *P[en]* and multiple proteins bound to the genomic *en/inv* region and that chromatin structure also plays a role. These interactions cause *P[en]* to be concentrated in the vicinity of *en/inv* and then transposition occurs into *en/inv* and genes nearby.

Fig. 5 shows a model for P-element homing. We suggest that *P[en]* homing is mediated by the interaction of multiple proteins bound to the *en* fragment within *P[en]* and proteins bound to the *en* genomic region, and that these interactions are facilitated by the H3K27me3 mark characteristic of PcG target genes. Note that since *P[en]* insertions into the *en/inv* target occur much more frequently than into other PcG-target genes, protein-protein interactions, specific for the *en/inv* region must be involved in homing. The smallest fragment that could mediate homing was 1.6kb, a size capable of binding many proteins. This suggests that *P[en]* homing is not caused by a binding of a single protein or protein complex. However, it is also possible that the 1.6 kb fragment is needed to form the chromatin structure that facilitates homing. Finally, we suggest that *P[en]* interacts with multiple proteins bound to the *en/inv* domain, since homing still occurs in *en^Δ1.5^*, where the majority of the genomic homing fragment has been deleted.

P-element homing occurs in germ cells. En is not expressed in these cells. Recent results indicate that the H3K27me3 modification is present at many developmental loci in germ cells (reviewed in [34]). *P[en]* homing suggests that, in addition to the H3K27me marks, there are specific proteins bound to *en* DNA in the germ cells. These proteins could be present to keep *en* silenced, or perhaps they are there to facilitate rapid initiation of *en* transcription in the embryo.

## Materials and Methods

### Construction of plasmids

*P[enHSP]* constructs were generated by inserting PCR fragments with *Spe*I and *Not*I ends into *pUZ* [23]. Inserts were generated by PCR with the following *en* primers. *P[enHSP1]* (−2.407 to −0.395 kb): P1-GGGGCGGCCGCGAATTCCGTTGATATGAT and P2-GCGACTAGTGCATGCTGGAGCTGTCAG; *P[enHSP2]* (-1.945 to -0.395kb): P3-GCGGCCGCGAAAGTGTGTAGGGGAAT and P2; *P[enHSP3]* (-2.407 to -0.579kb): P1 and GCGACTAGTCCACAGACACTTTTC [12]; *P[enHSP4]* (-1.945 to -0.847kb) P3 and GCGACTAGTGAGGCCTTCAATTAACCA; *P[enHSP5]* (-1.495 to -0.395kb): GCGGCCGCGCGCATAAAAATTGA and P2; *P[enHSP6]* (-0.576 to -0.395kb): GCGGCGGCCGCGAGATGGCATGTGGCTCTC and P2; *P[enHSP7]* (-0.941 to -0.415kb): GCGGCGGCCGCCGATGGGCAATATAAATTAAATG and GCGACTAGTGGTTGACAACTGTGTCCCCAGCG. The PCR-amplified fragments were cut with both *Spe*1 and *Not*1 and ligated into *pUZ*. The resulting clones were sequenced in and around the cloned PCR fragment to ensure sequence fidelity.

### Transgenic lines

*P[enHSP]* lines were generated by injections into *w^1118^* embryos by Genetic Services (Sudbury, MA, USA). For *P[enHSP]* inserts that mapped to chromosome 2, recombination mapping was used to determine whether they were in the vicinity of *en*. For *P[en3R]* mobilization, *P[en3R]-87kb* or *P[en3R]+52kb CyO/Sp* or *en^Δ1.5^; P(Δ2,3)99B Sb ry^506^/ry^506^* males were crossed to *ry^506^* females. *ry^+^* non-*CyO* progeny represented transposition events. All *P[en3R]* insertions on the second chromosome were localized by inverse PCR as described [12]. For *P[walter]-48A* mobilization, w*; P[walter]-48A/Sp or *CyO*; *P(Δ2,3)99B Sb ry^506^/+* males were crossed to *y Df(1)w67c2* females and *w^+^ Sp* or *CyO* males were selected and mapped with respect to chromosome. Insertions sites on the *CyO* chromosome were determined by inverse PCR [12]. Insertions on the *Sp* chromosome were mapped via recombination mapping to determine whether they were tightly linked with *en*.

### Generation of *en^Δ1.5^*

*en^Δ1.5^* was obtained in an experiment designed to recover a gene conversion event replacing wild-type *en* DNA (Fig. S1). It arose as the result of an imprecise excision of a P-element inserted 412bp upstream of the major transcription start site of *en*. The primer sequences used to characterize the deletion are: 1F: CAGTGCGACAATTGAGTTG, 1R: GCTTGTTAGGCAGCAAT, 2F:GGAAAGTGTGTAGGGGAAT, 2R:GAATCTGTTCGATGTGA, 3F:TCACATCGAACAGATTCG, 3R: ATCGATTTGCCAGACGAG. 4F: TTCAAGTCCATTGATC. The *en^Δ1.5^* deletion includes 1462 bp of genomic sequence from 7415804 to 7417265 (genome release 5.36), and it leaves 32bp (CATGATGAAATTATGTTAATAACATAATAATTA) in this genomic location.

## Supporting Information

Figure S1 **Generation of** ***en^Δ1.5^*** **.** (A) *en^Δ1.5^* was generated by a crossing scheme designed to obtain a gene conversion event by P-element excision [26]. The recipient chromosome was marked with *Sp* and contained a *ry+* P-element inserted 412 bp upstream of the *en* transcription start site. The donor chromosome contained a 16kb deletion of *en* DNA (*en^ΔJ83B^*) from −15 kb upstream through the first intron of *en* (generated in our lab), and the donor P-element that contained LoxP sites and FRT sites flanking (indicated by vertical lines in the *en* DNA) the PREs (P[en2] from [18]). We were trying to get a gene conversion event that would put LoxP and FRT sites into the genome. We set up 400 vials of Cross 1 and 300 vials of Cross 2. From this, we obtained 2 potential deletions (*en^Δ1.5^* and one other) and one potential gene conversion event. The 2 deletions were recovered and balanced from Cross 3 but the gene conversion event was not recovered. (B) Schematic representation of the PCR reactions used to detect the gene conversion event. L(LoxP), F(FRT). A, B are approximate primer locations.
(TIF)
