# Supplementary figures and images for: P-Element Homing Is Facilitated by engrailed Polycomb-Group Response Elements in Drosophila melanogaster
Source: PLoS One. 2012 Jan 19;7(1):e30437. doi: 10.1371/journal.pone.0030437 (PMC3261919; doi:10.1371/journal.pone.0030437)

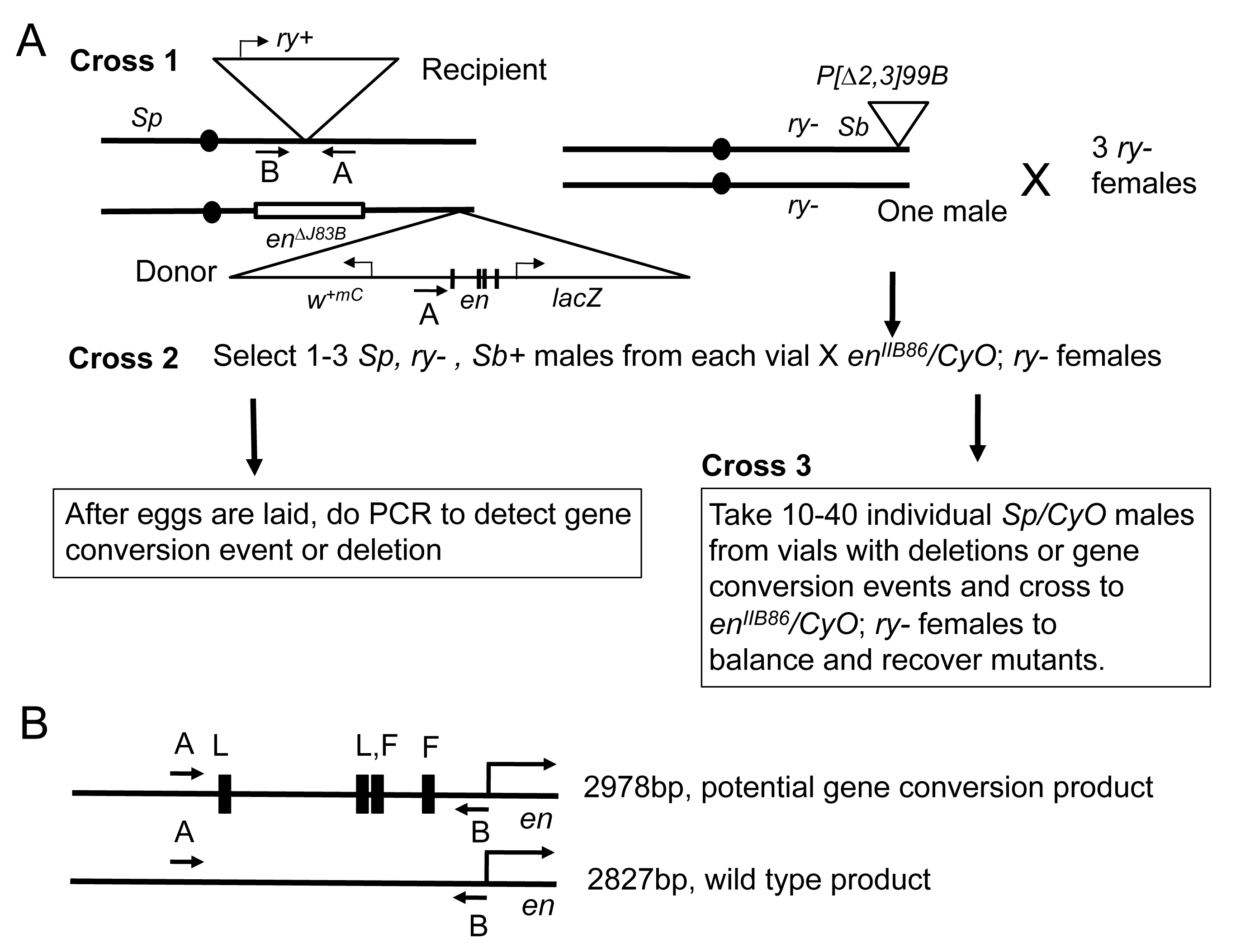

Supplement: Figure S1 — Generation of enΔ1.5 . (A) enΔ1.5 was generated by a crossing scheme designed to obtain a gene conversion event by P-element excision [26]. The recipient chromosome was marked with Sp and contained a ry+ P-element inserted 412 bp upstream of the en transcription start site. The donor chromosome contained a 16kb deletion of en DNA (enΔJ83B) from −15 kb upstream through the first intron of en (generated in our lab), and the donor P-element that contained LoxP sites and FRT sites flanking (indicated by vertical lines in the en DNA) the PREs (P[en2] from [18]). We were trying to get a gene conversion event that would put LoxP and FRT sites into the genome. We set up 400 vials of Cross 1 and 300 vials of Cross 2. From this, we obtained 2 potential deletions (enΔ1.5 and one other) and one potential gene conversion event. The 2 deletions were recovered and balanced from Cross 3 but the gene conversion event was not recovered. (B) Schematic representation of the PCR reactions used to detect the gene conversion event. L(LoxP), F(FRT). A, B are approximate primer locations. (TIF) [file pone.0030437.s001.tif]
